# Stroke in Birjand, Iran: A Hospital-Based Study of Acute Stroke

**DOI:** 10.5812/ircmj.4282

**Published:** 2013-03-05

**Authors:** Mohammad Dehghani Firoozabadi, Toba Kazemi, Gholamreza Sharifzadeh, Somayeh Dadbeh, Parvaneh Dehghan

**Affiliations:** 1 Birjand University of Medical Sciences Birjand, IR Iran; 2 Birjand Atherosclerosis and Coronary Artery Research Center, Birjand University of Medical Science, Birjand, IR Iran; 3 Student Research Committee, Birjand University of Medical Science, Birjand, IR Iran

**Keywords:** Stroke, Risk Factors, Hospital Mortality, Iran

## Abstract

**Background:**

Stroke, or cerebrovascular accident (CVA), is the second leading cause of death in the world and based on the World Health Organization (WHO) report in 2006, it is responsible for 9.9% of all deaths in the world which over 85% of these deaths occurred in developing countries.

**Objectives:**

The aim of this study was to investigate the data related to the frequency, risk factors, types and mortality of stroke in Birjand city.

**Patients and Methods:**

A retrospective cohort of consecutive patients with diagnosed stroke who were admitted to hospital (the only neurological center of Birjand) between 2002 and 2008 was designed. A stroke was defined according to clinical features and CT-scan which was confirmed by agreement of a staff neurologist. Collected data included date of admission, age, sex, and hospital outcome as well as related risk factors.

**Results:**

Totally, 1219 stroke (85.4% ischemic type) with the mean age of 69.6 ± 12.9 years and female: male ratio of 1.09 was included in over 6 years. The stroke hospital admission rates were 48.6 and 103.4 /100,000 population /year in the first and the last year of the study, respectively. There was an increasing trend in stroke incidence rate during the study (P < 0.01). The most common epidemiological risk factors for stroke in our region were hypertension, cardiac diseases, a history of stroke, diabetes mellitus, dyslipidemia, and smoking (54.7, 24.4, 20.1, 14.9, 12.2, and 9%, respectively). Overall in-hospital mortality rate was 17.1%.

**Conclusions:**

The stroke hospital admission rate might be increasing in Birjand. Therefore, health care administrators and public health authorities must work harder to promote the knowledge and practice of society about the stroke related risk factors and prevention methods.

## 1. Background

Stroke, or cerebrovascular accident (CVA), is the second leading cause of death in the world and based on the World Health Organization (WHO) report in 2006, it is responsible for 9.9% of all deaths in the world which over 85% of these deaths occurred in developing countries (1). Several studies has been performed about changes in incidence of stroke and its related mortality rate in different parts of the world, and generally showed gradual but remarkable decrease in the stroke mortality rate during recent decades (2, 3) However, according to WHO, changes in stroke mortality in most populations were mainly due to changes in case fatality rather than changes in event rates (4) which can reflect changes in the management of stroke or changes in disease severity. Moreover, some studies indicated the increased use of preventive treatments and major reductions in premorbid risk factors among the populations as a reason of these changes in stroke (5). So that due to improved diagnosis, treatment and control of hypertension (HTN) and other vascular risk factors has decreased stroke mortality rate as well as cardiac infarction (6, 7). The data about stroke varies from country to country, and recent studies have investigated to clarify this variations (8). A systematic review by Hosseini et al. (9) indicated that several studies published in two past decades showed the annual stroke incidence of various ages in Iran ranged from 23 to 103 per 100,000 population. Birjand, the administrative center of the Southern Khorasan province, is located in eastern part of Iran with a total population of 240,894 (70.6% lives in urban area). Previous two studies by Ghandehari et al. (10, 11) indicated the incidence of ischemic stroke in this population (all across the province) was 43.17 cases per 100,000 people per year. As the stroke mortality rate is an important health status indicator of a population, close observation of stroke cause of death as well as related risk factors provide more information in different populations.

## 2. Objectives

The aim of this study was to investigate the data related to the frequency, risk factors, types and mortality of stroke in Birjand city.

## 3. Patients and Methods

This retrospective cohort study included all consecutive admissions to Vali-e-Asr Hospital of Birjand Medical University, a tertiary care facility (the only neurology unit in the entire city), between March 2002 and July 2008, who had a confirmed CVA diagnosis. Every hospital admission was considered as a separate event and thus, patients may have been registered for more than one record. A database that collected patients’ information was used to identify eligible patients. Further data that did not exist in the database were gathered by researcher via evaluation of patient records. This study was approved by the institutional review board of the Birjand University of Medical Sciences, Birjand, Iran. A waiver of consent was granted given the retrospective nature of the project. Inclusion criterion was patients with a diagnosed acute CVA. The diagnosis of CVA was based on the clinical features related with neuroimaging data (brain CT scan) which was confirmed by agreement of a staff neurologist. Stroke was defined based on WHO criteria as an episode of relevant focal deficits with acute onset, documented by neurological examination, and lasting for more than 24 hours. Patients with a transient ischemic attack (TIA) or patients referred from other cities were excluded. The encoded data included demographic data, type of CVA (ischemic or hemorrhagic), occurrence of in-hospital death and stroke risk factors i.e. HTN, diabetes mellitus (DM), dyslipidemia, cardiac disease, smoking status, and previous history of CVA. Results are given as “means ± standard deviation” for continuous variables and number (percent) for categorical variables. The chi-square test or Fisher’s exact test was used for categorical variables and the Students t-test or the Wilcoxon Rank Sum test for continuous variables. Statistical analyses were performed using SPSS v.18 (SPSS, Chicago, Illinois, USA) and a p value less than 0.05 was considered significant.

## 4. Results

A total of 1219 patients with stroke were included in our cohort study. Patients’ demographic data is showed in Table 1. Six hundred thirty six patients (52.2%) were women. The mean age of patients was 69.6 ± 12.9 years with a range between 19 and 102 years and 72.4% of patients were older than 65 years. Totally, 642 patients (52.7%) lived in rural area. The stroke hospital admission rates were 48.6, 70.2, 78, 80.9, 98.4, and 103.4 per 100,000 Birjand inhabitants respectively in first six year of the study. There was a significant increase in the number of stroke admission during the study period (P < 0.01) which the incidence rate was twice as high after six years. Also, stroke subtypes are shown in Table 1; 85.4% of stroke events were of the ischemic type. Among ischemic subtypes, lacunar stroke was found in 93 patients (8.9%). Intracerebral hemorrhagic stroke (ICH) was the most frequent of hemorrhagic subtypes with a rate of 41.5% (n = 73). There was no significant difference in type of stroke between men and women (P = 0.98), different age groups (P = 0.14), and the living area (P = 0.77). HTN was the most important risk factor in 54.7% of stroke patients, which was followed by cardiac disease (24.4%), a history of stroke (20.1%), DM (14.9%), dyslipidemia (12.2%), and smoking (9%). Totally, 25% of patients did not have any known risk factor and 36.1% have at least one risk factor. The mean number of risk factors per person was 1.35 ± 1.1 in our patients. The mean number of risk factors per person was similar in ischemic and hemorrhagic types or men and women, but it was significantly correlated with age (P < 0.001). There was a significant difference between ischemic and hemorrhagic stroke in the presence of cardiac disease (25.6 vs. 18%, P = 0.03), DM (15.8 vs. 10.1%, P = 0.05), and dyslipidemia (12.8 vs. 9%, P = 0.05) (Table 2). The rate of in-hospital death was 17.1% (107 men and 101 women died during the study). Although the number of stroke patients significantly increased during years of the study, there was no significant change in the rate of in-hospital stroke death (P = 0.2). Most of the stroke deaths occurred in over-65 age group (81.2%) and there was a significant correlation between age and the mortality rate (P < 0.001). The rate of death was significantly higher in ischemic stroke (14.5 vs. 5.5%, P < 0.001).

**Table 1.** Demographic Dataand Hospital Outcome of Stroke Patients

	Stroke		Total
	Ischemic	Hemorrhagic	
**Number, No. (%)**	1041 (85.4)	178 (14.6)	1219 (100)
**Gender, No. (%)**			
Male	498 (85.4)	85 (14.6)	583 (47.8)
Female	543 (85.4)	93 (14.6)	636 (52.2)
**Age group, No. (%)**			
15-45	51 (92.7)	4 (3.7)	55 (4.5)
46-65	233 (82.9)	48 (17.1)	281 (23.1)
> 66	757 (85.7)	126 (14.3)	883 (72.4)
**Mean age, y, Mean ± SD**	69.8 ± 13.6	68.8 ± 12.1	69.6 ± 12.9
**Location, No. (%)**			
Urban	488 (84.6)	89 (15.4)	577 (47.3)
Rural	553 (86.1)	89 (13.9)	642 (52.7)
**Hospital outcome, No. (%)**			
Death	151 (72.6)	57 (27.4)	208 (17.1)
Discharge	890 (88)	121 (12)	1011 (82.9)

**Table 2.** Age, sex, andSubtype Distributions of Stroke Risk Factor

	Risk Factors					
	Hypertension	Diabetes Mellitus	Dyslipidemia	Cardiac disease	Cigarette	Previous stroke
**Gender, No. (%)**						
Male	266 (45.6)	78 (13.4)	71 (12.2)	139 (23.8)	82 (14.1)	127 (21.8)
Female	401 (63.1)	104 (16.4)	78 (12.3)	159 (25)	28 (4.4)	118 (18.6)
**P value**	< 0.001	0.15	0.96	0.6	< 0.001	0.16
**Age group, No. (%)**						
15-45	12 (21.8)	4 (7.3)	6 (10.9)	11 (20)	9 (16.4)	5 (9.1)
46-65	135 (48)	55 (19.6)	52 (18.5)	70 (24.9)	35 (12.5)	57 (20.3)
> 66	520 (58.9)	123 (13.9)	91 (10.3)	217 (24.6)	66 (7.5)	183 (20.7)
**P value**	< 0.001	0.02	< 0.001	0.7	< 0.001	0.11
**Stroke Subtypes**						
Ischemic	561 (53.9)	164 (15.8)	133 (12.8)	266 (25.6)	97 (9.3)	213 (20.5)
Hemorrhagic	106 (59.6)	18 (10.1)	16 (9)	32 (18)	13 (7.3)	32 (18)
**P value**	0.16	0.05	0.05	0.03	0.39	0.45
**Total**	667 (54.7)	182 (14.9)	149 (12.2)	298 (24.4)	110 (9)	245 (20.1)

## 5. Discussion

Our study reflected the stroke data in Southern Khorasan province and its changes over six years. Some previous studies in this region indicated the mean of annual incidence rate of ischemic stroke during 2001 and 2005 was 43.17 cases per 100,000 people per year (11). This was similar to the primary years of our study, but the incident rate had an upward trend during the study period and was 103.4 cases per 100,000 people in 2008. It means the stroke hospital admission rate is increasing in Birjand, although this is in accordance with an increasing trend in stroke incidence in other regions of Iran (12) or other developing countries(13). On the other hand this increasing rate can reflect an improvement in awareness of people about CVA and its signs or symptoms as well as more feasible and facile accessibility to health care centers, which both can raise the number of referred patient to hospitals. Further studies can investigate the possible true increasing stroke rate in this region. In our study ischemic stroke was the majority of stroke subtypes and about 85% of our patients presented it. This rate is in the range of other comparable studies in developing or developed countries (14). Our patients had a mean age of about 70 years with a female: male ratio about 1, which was similar to other Iranian studies (12). Gender and age distributions among our patients were more similar to what has been reported in developed countries: in Fonarow et al. study, the mean age of patients was 70.1 years, and females were affected nearly as frequently as males (53.5%) (15). In regards to risk factors of ischemic stroke, HTN was the most common risk factor, as expected, followed by cardiac disease, history of stroke, diabetes, dyslipidemia and smoking, respectively. This figure is seen in other parts of Iran (16) as well as other countries neighboring the Persian Gulf (17, 18) This pattern of highly prevalent risk factors is also similar to the other developing countries (19, 20) and indicates the importance of effective interventions for prevention and treatment of these potentially fatal risk factors among target population to reduce the burden of CVA and other related diseases and morbidities. Consequently, in-hospital mortality rate among the stroke patients was 17.1%. There was no significant change in in-hospital mortality rate, observed from 2002 to 2008 among our stroke patients, despite the fact that the incidence of stroke was significantly increased during these years. Although the rate of in-hospital death in our study is much higher than developed countries,(15) its stability in the years of study can indicate either an improvement of health care system for management of these patients or possible increasing in mild stroke hospital admission during the study period. A limitation of our research is the design of hospital-based study which should be improved in further community-based study in our area. In conclusion, this study demonstrated that the stroke hospital admission rate might be increasing in Birjand. Also, some features of the Birjand hospital based stroke study are more similar to developing countries (risk factors and mortality rate) and some others are more similar to developed countries (age and sex distribution and type of stroke). Therefore, health care administrators and public health authorities must work harder to promote the knowledge and practice of society about the stroke related risk factors and prevention methods. We suggest developing a stroke data bank and performing population-based longitudinal studies in all regions of the country.
